# Severe anti-NMDA receptor encephalitis in a pediatric patient presenting with status dystonicus as the primary clinical manifestation: A case report (CARE)

**DOI:** 10.1097/MD.0000000000046361

**Published:** 2025-12-12

**Authors:** Pingping Tian, Juan Li, Meng Wang, Wandong Hu, Xiufang Ding, Yong Liu, Hongwei Zhang

**Affiliations:** a Neurology Department, Children’s Hospital Affiliated to Shandong University, Jinan, Shandong, China; b Neurology Department, Jinan Children’s Hospital, Jinan, Shandong, China.

**Keywords:** anti-NMDAR encephalitis, children, Midazolam, severe, status dystonicus

## Abstract

**Rationale:**

Anti-NMDAR encephalitis is the most prevalent form of autoimmune encephalitis, and status dystonicus may occur during the acute phase. Status dystonicus is a severe and rare acute movement disorder characterized by clinical manifestations resembling those of status epilepticus. However, there are currently limited case reports regarding anti-NMDAR encephalitis complicated by status dystonicus in pediatric patients. In this study, a pediatric patient with severe anti-NMDAR encephalitis developed status dystonicus during the acute phase. Symptoms gradually resolved following continuous intravenous infusion of midazolam.

**Patient concerns:**

The patient was admitted to our hospital for detailed evaluation due to abnormal emotions, involuntary movements, and language impairment. Following admission, the patient developed seizures, impaired consciousness, cognitive dysfunction, and status dystonicus. Abnormal results were found in the electroencephalogram and cerebrospinal fluid tests, and the anti-NMDAR antibody was positive.

**Diagnoses:**

The child was diagnosed with severe anti-N-methyl-D-aspartate receptor encephalitis, presenting primarily with state dystonicus.

**Interventions:**

The child received immunotherapy including immunoglobulin, methylprednisolone pulse therapy, and rituximab. When status dystonicus occurred, continuous intravenous infusion of midazolam was administered, resulting in resolution of the symptom.

**Outcomes:**

Electroencephalography can effectively differentiate status dystonicus from status epileptic.Continuous intravenous infusion of midazolam can alleviate status dystonicus.

**Lessons:**

During the acute phase of anti-NMDAR encephalitis, pediatric patients may develop status dystonicus. Neurologists should promptly distinguish it from status epilepticus and implement appropriate management strategies to improve patient outcomes.

## 1. Introduction

Anti-NMDAR encephalitis is the most common form of autoimmune encephalitis (AE), accounting for 80% of all AE cases. It was first described by Dalmau et al in 2007.^[1]^ The incidence of this disease has been steadily increasing over the years. According to recent epidemiological data, the incidence rate is 0.17 per 100,000.^[2]^ Clinically, it typically presents with acute or subacute onset of epileptic seizures, mental and behavioral disturbances, cognitive dysfunction, abnormal movements, and impaired consciousness. In some pediatric patients, involuntary movements may intensify, progressing to status dystonicus. Status dystonicus (SD) is a severe and rare acute movement disorder characterized by clinical manifestations resembling those of status epilepticus (SE), with a mortality rate as high as 12.5%.^[3]^ Early recognition and timely intervention are critical for improving patient outcomes. However, there are currently limited case reports regarding anti-NMDAR encephalitis complicated by status dystonicus in pediatric patients, suggesting that clinicians have an insufficient level of awareness and understanding of this condition. Therefore, this article presents a case of severe anti-NMDA receptor encephalitis in a pediatric patient with status dystonicus as the primary clinical manifestation. Based on a review of previous literature, this study analyzes the etiological factors, clinical features, treatment strategies, and prognosis of the case, aiming to enhance clinicians’ understanding of this subtype of anti-NMDAR encephalitis.

## 2. Case presentation

This study was approved by the institutional review board of the authors. Informed consent was obtained from the patient for publication of this case report details.

### 2.1. Retrospective studies

**Figure 1.**
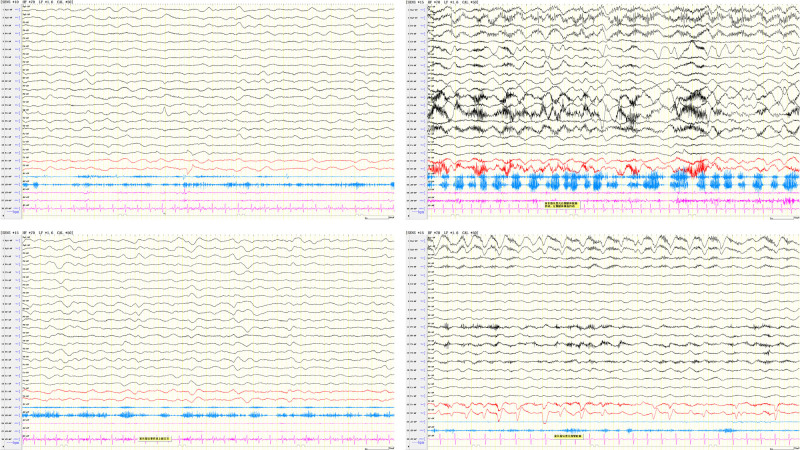
When the child had status dystonicus, the EEG showed: (A) background activity – diffuse slow wave activity (δ); interictal discharges: consciousness disorder – sharp-slow waves of 2 to 3Hz in the right middle temporal area; (B–D) indicated that the child had crossed movements of both upper limbs or choreiform movements of the limbs or blinking, without ictal patterns at the same time, accompanied by movement artifacts. The nature of the episode was qualitatively determined to be a non-epileptic event. EEG = electroencephalographic.

The patient is an 8-year-old boy who presented to Shandong University Children’s Hospital with a chief complaint of “abnormal mood for 5 days, followed by the onset of speech disorders and involuntary movements for 2 days.” Five days prior to admission, the child experienced a fall and vomiting during play, and subsequently developing abnormal emotional, primarily characterized by irritability. Two days prior to admission, the child developed speech impairment and involuntary movements, presenting as incoherent speech, slurred articulation, involuntary head shaking, and flailing limbs. During this period, the child exhibited impaired recognition of individuals, confusion, and sleep disturbances. The child had previously been in good health. No significant abnormalities were noted in his personal and family medical history. Physical examination upon admission: temperature (T) 36°C, pulse (P) 86 beats/min, respiratory rate (R) 18 breaths/min, weight (WT) 38.5 kg, and blood pressure 115/70 mm Hg. The patient presented with confusion (Glasgow coma scale 4), poor responsiveness, and uncooperativeness during the physical examination. The Babinski sign was positive on the right side and negative on the left side. Following admission, the child experienced progressively worsening impaired consciousness, gradually developing an inability to speak, involuntary movements of the limbs, worsening sleep disturbances, and frequent seizures. Brain magnetic resonance imaging revealed no significant abnormalities. Cerebrospinal fluid analysis revealed a white blood cell count of 26 × 10⁶/L. Testing for serum and cerebrospinal fluid AE antibodies demonstrated positivity for anti-glutamate receptor (NMDA-type) IgG antibody at a titer of 1:1000. Electroencephalogram (EEG) findings were abnormal: background activity showed significant slowing compared with age-matched norms, with occasional theta rhythm observed in the occipital region and diffuse slow wave activity. Seizure events: Electroclinical seizures were recorded, including 1 episode of focal seizure accompanied by impaired consciousness. A diagnosis of anti-NMDAR encephalitis was established, and the child received high-dose intravenous immunoglobulin combined with methylprednisolone pulse treatment. During the hospitalization, lacosamide and levetiracetam were sequentially administered to manage convulsive seizures. Aripiprazole (5 mg/d) and clonazepam (2 mg/d) were prescribed to address psychiatric symptoms and sleep disturbances. The child’s involuntary movements progressively worsened, primarily characterized by frequent blinking, bilateral upper limb crossing, or flailing of all 4 limbs, accompanied by increased muscle tone. EEG examination revealed diffuse slow wave activity, interictal epileptiform discharges, and 2 to 3 Hz sharp-slow wave complexes in the right middle temporal region, with no seizure-related patterns identified and the presence of movement-related artifacts (as shown in Fig. 1), indicating that the abnormal movement is not an epileptic seizure.The child received oral trihexyphenidyl (4 mg/d) to ameliorate abnormal movements and alleviate muscular rigidity. However, no improvement in symptoms was observed. The dosage of trihexyphenidyl was subsequently increased to 6 mg/d, yet there has been no significant improvement in the patient’s condition. On the 29th day of hospitalization, the child exhibited marked agitation and restlessness, accompanied by limb stiffness and tremors, elevated muscle tone, and excessive diaphoresis. These symptoms persisted without relief, leading to a diagnosis of status dystonicus. The child was sequentially administered chloral hydrate enema; intramuscular phenobarbital, and intravenous diazepam; however, no significant improvement was observed. Continuous intravenous infusion of midazolam (1 µg/kg/min) was administered as maintenance therapy, with the dosage dynamically adjusted to a maximum of 3.5 µg/kg/min. After 12 days of treatment, the child’s irritability was significantly alleviated compared with baseline, and dystonia showed marked improvement. Following dose reduction and discontinuation of midazolam, olanzapine (2.5 mg/d) was initiated as maintenance therapy. Despite receiving methylprednisolone pulse therapy and 2 courses of intravenous immunoglobulin treatment, the child remained unconscious and in critical condition, with an modified Rankin Scale persistently ≥ 4. Subsequently, after communicating with the child’s parents, 4 doses of rituximab were administered as second-line therapy, following which the child’s level of consciousness progressively improved. The patient was hospitalized in the neurology ward for 58 days before being transferred to the rehabilitation department for rehabilitation treatment. Following 4 months of comprehensive rehabilitation treatment, the child’s cognitive function, language ability, and motor skills had largely recovered to pre-illness levels. The clinical course and medication history of the pediatric patient are detailed in Tables 1 and 2.

**Table 1:** Clinical course of the child’s condition.

Timeline	Clinical manifestations and disease progression	Diagnosis and Clinical Assessment	Interventions
Hospital days 1–3	Altered consciousness, speech impairment, involuntary movements, convulsive seizures, and sleep disturbances	Diagnosis of anti-NMDAR encephalitis	Initiated first-line immunotherapy (IVIG/Corticosteroids) and symptomatic treatment
Hospital day 6	Fever	Diagnosis of hypostatic pneumonia	Initiated antibiotic therapy
Hospital days 19–29	Gradual worsening of involuntary movements		Started oral Trihexyphenidyl treatment
Hospital day 29	Rigid shaking of limbs, increased muscle tone, agitation, profuse sweating, and persistent without relief	Diagnosis of status dystonic	Initiated continuous intravenous infusion of Midazolam
After 12 d of Midazolam	Reduced involuntary movements, decreased irritability, and improved muscle tone.		Tapered and discontinued Midazolam; added Olanzapine
Hospital day 30	Remained unconscious, mRS score ≥ 4	Poor response to first-line immunotherapy	Initiated second-line immunotherapy (Rituximab)
Hospital day 58	Clinical improvement		Transferred to Rehabilitation Department for rehabilitation therapy
After 4 mo of rehab	Cognitive, language, and motor functions have returned to baseline	Favorable prognosis	

IVIG = intravenous immunoglobulin, mRS = modified Rankin Scale.

**Table 2:** Medication details for the child.

Treatment Category	Medication	Dose	Duration	Response	Side effects
Immunotherapy	IVIG	Total dose: 2 g/kg, administered over 3 d	Repeatd the same dose of medication after a 3-wk interval	Poor initial response	None
	Methylprednisolone	700 mg/d × 3 d, 350 mg/d × 3 d, 170 mg/d × 3 d, followed by low-dose maintenance	Started on hospital day 3	Poor initial response	None
	Rituximab	428 mg/dose, for a total of 4 doses at 1-wk intervals	Started in week 5, over 4 wk	Effective, clinical improvement	None
Antiseizure Treatment	Lacosamide	2–3.2 mg/kg/d	11 d	Effective	Suspected allergic reaction
	Levetiracetam	700 mg/d	44 d	Effective	None
Psychiatric Symptoms/Sleep Disorder	Aripiprazole	5 mg/d	57 d	Effective	None
	Clonazepam	1–2 mg/d	51 d	Effective	None
Movement Disorder	Trihexyphenidyl	4–6 mg/d	40 d	Ineffective	None
	Midazolam	1–3.5 µg/kg/min	12 d	Effective	None
	Olanzapine	2.5 mg/day	Started after Midazolam discontinuation, for 18 d	Effective	None

IVIG = intravenous immunoglobulin.

## 3. Discussion

Anti-NMDAR encephalitis is an autoimmune-mediated inflammatory disorder characterized by clinical heterogeneity, presenting with a complex array of manifestations including epileptic seizures, psychiatric and behavioral disturbances, movement disorders, and alterations in consciousness. Nearly half of affected children may develop movement disorders,^[4]^ typically manifesting as repetitive and stereotyped movements of the orofacial region or limbs, such as perioral twitching, chewing-like motions, and choreiform limb movements. If these movement disorders persist without resolution, life-threatening complications such as hyperthermia, electrolyte disturbances, rhabdomyolysis, and renal failure may arise, potentially progressing to status dystonicus. SD is also referred to as dystonic crisis or dystonic storm,^[3]^ It was initially described by Jankovic et al in 1982^[5]^ and is defined as a rapidly progressive, abnormally frequent, and severe generalized dystonic episode.^[6]^ The incidence in adult patients with anti-NMDAR encephalitis is ~ 8%,^[4,7]^ with limited data available for children. The clinical presentation closely resembles that of SE, making it susceptible to misdiagnosis and underrecognition.

Lumsden et al^[8]^ proposed a 5-stage progression of dystonia severity. In the second and third stages, patients experience gradually worsening dystonia compared with baseline levels, but without life-threatening complications. However, if timely intervention is not administered, the condition may progressively deteriorate into the fourth and fifth stages, where status dystonicus develops and requires urgent intervention. This pediatric patient presented with persistent involuntary movements accompanied by dystonia. A simultaneous EEG was conducted, revealing no evidence of epileptic seizures. Given the concurrent noninfectious fever, the child was assessed as Grade IV according to the DSAP rating scale^[8]^ and diagnosed with status dystonicus. If clinicians lack sufficient understanding of status dystonicus, it is highly prone to misdiagnosis as SE. SE is defined as either intermittent convulsive seizures with loss of consciousness during the interictal period or continuous convulsive activity lasting at least 30 minutes without relief, accompanied by epileptiform discharges on EEG within this timeframe. In contrast, status dystonicus represents a manifestation of extrapyramidal system involvement. Its diagnostic criteria primarily depend on the severity of clinical symptoms and the presence of complications, without specific temporal requirements. Notably, epileptiform discharges are typically absent on EEG during this condition, which may be triggered or exacerbated by external stimuli. Therefore, electroencephalography is the optimal method for early diagnosis and differential diagnosis of status dystonicus.

The pathogenesis of status dystonicus in anti-NMDAR encephalitis remains unclear. It may be associated with alterations in dopaminergic pathways within the cortex and subcortical regions, including the brainstem and basal ganglia, resulting from widespread cerebral dysfunction.^[4,7]^ SD is predominantly observed in children and adolescents,^[9–14]^ with a higher prevalence among males (64.7%).^[12]^ The underlying etiology is thought to be potentially associated with cerebral immaturity.^[9,15]^ This case involved an 8-year-old boy, consistent with previous studies. SD is categorized into tonic and phasic subtypes based on clinical features. The phasic subtype accounts for approximately one-third of all cases and is more frequently observed in females and in secondary status dystonicus.^[12]^ It is associated with a more favorable prognosis compared with the tonic subtype.^[10]^ This child primarily presented with irritability and tonic tremors in the limbs, which corresponds to the phasic subtype classification. Research has found^[7]^ that anti-NMDAR antibodies in the cerebrospinal fluid are universally strongly positive in anti-NMDAR encephalitis patients presenting with status dystonicus. Moreover, the incidence of ovarian teratoma is significantly higher among female patients with status dystonicus compared with those without status dystonicus. In this pediatric case, the anti-NMDAR antibodies in both the cerebrospinal fluid and serum were strongly positive at 1:1000, consistent with previous studies. This suggests that clinicians should be vigilant for status dystonicus in pediatric anti-NMDAR encephalitis with strongly positive anti-NMDAR antibodies while also screening for tumors in female patients with status dystonicus.

For pediatric patients with anti-NMDAR encephalitis complicated by status dystonicus, the fundamental treatment principle involves the activae Immunotherapy and timely implementation of sedative measures.^[16,17]^ Benzodiazepines are the most widely used sedative agents. In severe cases, intravenous infusion of midazolam is currently considered the cornerstone of pharmacological treatment due to its rapid onset and reliable efficacy. The duration of sedation therapy is determined based on the individual clinical response.^[3,7]^ In this case, following the onset of status dystonicus, midazolam was administered via intravenous infusion alongside treatment for the underlying condition. The child gradually entered a state of sustained drug-induced sleep, which effectively terminated the status dystonicus and halted its progression. Midazolam was gradually tapered and discontinued; oral olanzapine was initiated as maintenance therapy, resulting in favorable clinical outcomes. Children with anti-NMDAR encephalitis complicated by status dystonicus typically exhibit prolonged recovery times and unfavorable short-term prognosis; however, the long-term prognosis may remain unaffected.^[7]^ Currently, there is a lack of large-sample, prospective, controlled studies in this field.

## 4. Conclusion

In summary, status dystonicus in pediatric anti-NMDAR encephalitis is a rapidly progressive and life-threatening condition that is frequently misdiagnosed as SE. Video EEG monitoring is recommended for differential diagnosis. Aggressive management, including sedatives such as midazolam for severe cases, is essential. Despite limited evidence, early recognition and timely intervention are pivotal for improving prognosis.

### 4.1. Family perspective

This illness not only affected our child but also profoundly impacted our entire family. Our daily lives were brought to a halt. We took turns staying overnight at the hospital and struggled to explain to his siblings why their brother was behaving so unusually. The physical and emotional exhaustion we endured was overwhelming. The support provided by the neurology team became our cornerstone during this difficult time. Witnessing the dedication of the doctors and nurses as they worked tirelessly to save our child gave us the strength to persevere. This experience has deepened our understanding of the challenges associated with diagnosing and treating rare diseases. We hope that by sharing our story, we can raise greater awareness about such conditions and help other families feel less isolated in their own battles with illness.

## Acknowledgments

The authors thank the parents of the children involved in this study and contributors to the study who are not included in the author list.

## Author contributions

**Data curation:** Wandong Hu.

**Investigation:** Juan Li.

**Methodology:** Xiufang Ding.

**Software:** Meng Wang.

**Writing – original draft:** Pingping Tian.

**Writing – review & editing:** Yong Liu, Hongwei Zhang.
